# Bioassay-Guided Skin-Beneficial Effects of Fractionated *Sideritis raeseri* subsp. *raeseri* Extract

**DOI:** 10.3390/plants11202677

**Published:** 2022-10-11

**Authors:** Nemanja Krgović, Miloš Jovanović, Ana Alimpić Aradski, Teodora Janković, Tatjana Stević, Gordana Zdunić, Sonja Duletić Laušević, Katarina Šavikin

**Affiliations:** 1 Institute for Medicinal Plants Research “Dr. Josif Pančić”, Tadeuša Košćuška 1, 11000 Belgrade, Serbia; 2 Institute of Botany and Botanical Garden “Jevremovac”, Faculty of Biology, University of Belgrade, Takovska 43, 11000 Belgrade, Serbia

**Keywords:** ironwort, phenolics, antioxidant activity, antimicrobial activity, tyrosinase inhibition, sun protection factor

## Abstract

This study aimed to evaluate the potentials for skin-beneficial effects of the hydroethanolic extract of the aerial parts of ironwort (*Sideritis raeseri* Boiss. & Heldr. subsp. *raeseri*) and its fractions (petroleum ether, ethyl acetate, n-butanol and water). For these purposes, chemical analysis and in vitro antioxidant, anti-tyrosinase and antimicrobial assays, as well as determination of sun protection factor (SPF), were performed. The highest total phenolic content and quantity of individual flavonoids, chlorogenic acid and verbascoside were determined in the n-butanol fraction, which is in line with results obtained for antioxidant activity. The greatest antimicrobial activity against *Staphylococcus aureus*, *Staphylococcus epidermidis* and *Pseudomonas aeruginosa*, the most common causative agents of the skin infections in humans, was exhibited by the ethyl acetate fraction. The strongest anti-tyrosinase activity was shown by the hydroethanolic extract (52.64% of inhibition at 100 μg/mL). Almost all tested samples showed photoprotective activity with SPF higher than 6 obtained at a low concentration of 0.1 mg/mL, which was more than 15 for the butanol fraction. These findings revealed that the hydroethanolic extract of *S. raeseri* aerial parts could be a valuable ingredient in the formulation of cosmetic and medicinal products intended to overcome various skin disorders.

## 1. Introduction

In recent decades, medicinal plants have been comprehensively investigated by scientific researchers, whose findings have proved their plentiful contents of natural biomolecules and support the use of plant extracts to improve health or to treat some specific diseases, particularly as a result of polypharmacological and synergistic effects [1,2]. The genus *Sideritis* L. (Lamiaceae) comprises more than 150 annual and perennial plants, mainly distributed in the Mediterranean region and in the moderate areas of Asia [3]. According to the results of numerous preliminary phytochemical studies of *Sideritis* species, terpenoids, flavonoids, phenolic acids, essential oils, iridoids, coumarins, lignans and sterols have been identified as the main constituents [4,5,6]. The aerial parts of *Sideritis* plants, also known as ironwort or mountain tea, prepared as an infusion or decoction, are widely used in folk medicine as anti-inflammatory, antiulcerative, antimicrobial, antispasmodic, analgetic and carminative agents [4]. Additionally, in various countries, *Sideritis* species are used topically for healing wounds and burns, and to stop the bleeding from a cut [3]. One of these plants, *Sideritis raeseri* Boiss. & Heldr. subsp. *raeseri*, is described in a relevant monograph of the European Medicines Agency, along with *S. scardica* Griseb, *S. clandestina* (Bory & Chaub.) Hayek and *S. syriaca* L., where it is stated that *S. herba*, in the form of herbal tea, can be orally used for the relief of cough associated with cold and for the relief of mild gastrointestinal discomfort through long-standing use [7].

Oxidative stress, a condition of cells or tissues, presented as an imbalance between the antioxidant capacities and production of ROS (reactive oxygen species), has an important role in the etiology of aging, including pigmentary skin disorders [8,9]. There is evidence from the research of Koutsianas et al. that an infusion obtained from a mixture of *S. perfoliate*, *S. raeseri* and *S. scardica* after topical cutaneous application exerted antioxidant, anti-aging, moisturizing and photoprotective properties [10]. On the other hand, the production of melanin is responsible for the pigmentation of the skin and is also a defense mechanism against harmful ultraviolet rays. In the case of excessive melanin production, some dermatological problems may occur [11]. Namely, tyrosinase is a pivotal enzyme involved in melanogenesis within melanocytes, and as such it is a potential target molecule for the treatment of dermatological conditions accompanied by skin hyperpigmentation. Since tyrosinase is a polyphenoloxidase, polyphenolic compounds can act as competitive enzyme inhibitors [12]. The use of plant extracts as a source of antioxidants and tyrosinase inhibitors could be one of the therapeutic approaches for the control of problems related to skin hyperpigmentation [11,13].

Infectious diseases are another problem affecting all human age groups, especially due to the microbial ability to form a biofilm on the skin surface, leading to drug tolerance of bacteria and the capacity to escape the host immune response [14]. Due to the rapid growth of antibiotic resistance, as well as more frequent opportunistic infections in immunocompromised patients, the need for alternative therapies with fewer side effects, such as the use of natural products from plant origin, is increasing [15]. Topical application of antimicrobial herbal extracts and preparations (creams, gels, lotions, soaps, etc.) to treat skin disorders caused by infectious microorganisms is in progress [16].

Additionally, ultraviolet (UV) irradiation is commonly considered to be a crucial causative reason for cutaneous photoaging, wherein both UVA and UVB light are involved in pro-aging molecular pathways. The harmful effects of UV radiation on the skin are reflected in the induction of free radical formation, DNA damage, inflammation, photoaging, immunosuppression, and the development of skin cancer. Natural photoprotective substances, such as plant secondary metabolites (phenylpropanoids and flavonoids), characterized by high photostability and low phototoxicity, are interesting candidates for the development of powerful photoprotective agents [17,18]. Many natural compounds that are absorbed in the UV spectrum can be successfully used as UV blockers. Thereby, plant extracts containing compounds that are effective UV absorbers can be used to formulate natural sunscreens [19].

Previous studies on *Sideritis* spp. have been focused mainly on elucidating bioactivities associated with traditional uses in gastrointestinal and respiratory disorders. However, there is a lack of studies examining the potential skin-beneficial effects of *Sideritis* extracts. Sato et al. (2022) [20] studied the anti-skin aging effect of *S. scardica* and three isolated flavonoids (isoscutellarein, 4′-o-methylisoscutellarein and 4′-o-methylhypolaetin). All of them possessed collagenase inhibition, inhibition of advanced glycation end product formation, and antiallergic activities. Moreover, 4′-o-methylisoscutellarein showed UVB-induced matrix metalloproteinase-1 expression inhibition activity. Moreover, some active compounds, such as verbascoside and chlorogenic acid, found also in *Sideritis* species, are potential active ingredients in pharmaceutical topical formulations and dermo-cosmetic products [21,22].

A key step for the systematic screening of the extract bioactivities and their correlation with the chemical composition is the separation and purification of the crude extract from the co-extracted ballast ingredients. For these purposes, bioassay-guided fractionation is a well-established approach. Accordingly, the aim of the present study was to determine total phenolic content, to quantify selected flavonoids abundant in *Sideritis* species and known for their potential skin-beneficial activities (derivatives of isoscutellarein and hypolaetin), chlorogenic acid and verbascoside by HPLC analysis, and to evaluate in vitro antioxidant, anti-tyrosinase and antimicrobial activity, as well as to determine the sun protection factor (SPF) of the hydroethanolic extract of the aerial parts of *S. raeseri* subsp. *raeseri* and its corresponding fractions.

## 2. Results and Discussion

### 2.1. Chemical Composition

The total phenolic content of *Sideritis raeseri* subsp. *raeseri* (SR) hydroethanolic extract and its fractions was in the range from 13.45 to 92.20 mg GAE/g (Table 1). The highest total phenolic content was determined in the n-butanol fraction, followed by the ethyl acetate fraction (88.48 mg GAE/g). The hydroethanolic extract and water fraction contained similar amounts (48.25 and 46.65 mg GAE/g, respectively), while the lowest total phenolic content was detected in the petroleum ether fraction. Regarding the content of individual compounds, all samples contained characteristic 8-hydroxyflavone 7-allosylglucosides (isoscutellarein and hypolaetin derivatives and their methyl ethers) widely distributed in the genus *Sideritis* [23,24]. The highest concentration of flavonoid glycosides ISC1, ISC2, HYP1 and HYP2 was recorded in the n-butanol fraction (27.14, 32.4, 26.47 and 11.9 mg/g, respectively). The ethyl acetate fraction also contained a higher amount of ISC2 (26.15 mg/g) and HYP2 (10.62 mg/g), whereas ISC1 and HYP1 were detected in higher amounts in the hydroethanolic extract and water fractions compared with the ethyl acetate fraction. The petroleum ether fraction was the least abundant in these compounds.

Apart from flavonoids, the main phenolic compounds found in the *Sideritis* species are hydroxycinnamic acid derivatives and phenylpropanoid glycosides, including chlorogenic acid and verbascoside, respectively [4,5]. Concentrations of chlorogenic acid and verbascoside were in the ranges of 0.21–2.85 and 1.62–31.01 mg/g, respectively. The distribution pattern of these compounds during re-extraction was similar to that for flavonoids, where the highest concentration of these comounds was found in the n-butanol fraction, followed by the ethyl acetate fraction and hydroethanolic extract.

Extraction is an important step for the recovery of valuable compounds from the plant material, and type and concentration of the starting extraction solvent is one of several factors that influence extraction efficiency. In our study, total phenolic content in hydroethanolic extract was similar to previously reported data for hydromethanolic (52.60 ± 8.75) and hydroethanolic (48.9 ± 0,3 mg GAE/g dw) extracts of the same species *S. raeseri* [25,26]. Polar solvents such as water or methanol have been shown to be more efficient than ethyl acetate in extracting total phenolics from *S. sipylea*, *S. perfoliata*, *S. ozturkii* and *S. arguta* [27,28,29,30]. However, it should be noted that differences in the extraction method may also affect the content of the components in the extracts. In all cited reports, plant material was extracted separately with different solvents, whereas our goal was to conduct successive extraction of the basic hydroethanolic extract by using solvents of different polarity in order to obtain fractions with chemical profile of active compounds according to their solubility. In that sense, the higher total phenolic content obtained in the ethyl acetate fraction in our study is in accordance with the report of Bardakci et al., who also performed successive extraction of *S. congesta* with various solvents and found higher total phenolic content in the ethyl acetate fraction compared to the methanolic extract or water fraction [31].

Our results for the content of individual phenolic compounds in hydroethanolic extract were slightly different compared with previously reported data for 96% ethanolic extract of *S. raeseri* [23]. Concentrations of HYP1 (2.89–5.31 mg/g dw) and ISC2 (0.79–2.24 mg/g dw) were much lower than in our study. On the other hand, in the study carried out by Šavikin et al. [32], the amounts of HYP1 (1.05–11.46 mg/g dw), ISC1 (0.68–10.68 mg/g dw) and ISC2 (0.74–15.56 mg/g dw), determined in hydroethanolic extract of the same species, were in agreement with our results. Although plant material in all studies was collected on the same mountain, diversity in the published results could be a consequence of genotypic and environmental variations, as well as the different solvents used for extraction, indicating that aqueous ethanol could be a preferable solvent system to methanol/water for the efficient extraction of bioactive compounds from *S. raeseri*.

The results obtained for chlorogenic acid and verbascoside content are in accordance with previous results. Erkan et al. determined higher chlorogenic acid content in methanol than in ethyl acetate extract of *S. congesta* (22.2 vs. 5.4 mg/g) and *S. arguta* (23.6 vs. 7.8 mg/g) [30], while Bardakci et al. determined higher verbascoside content in ethyl acetate (48% *w*/*w*) than in chloroform (1.27% *w/w*) extract of *S. congesta* [31].

### 2.2. Antioxidant Activity

The antioxidant activities of SR samples and positive controls were evaluated using four different in vitro assays (Table 2) in order to take various modes of antioxidant action into account. Hydroethanolic extract and its fractions were tested at three different concentrations (100, 200 and 500 μg/mL), while BHA, BHT and ascorbic acid (positive controls) were tested at a concentration of 100 μg/mL. In all assays, concentration-dependent antioxidant activity was noticed, with the highest extract concentration (500 μg/mL) being the most active. There is literature evidence that samples of different *Salvia* species tested at three concentrations also exhibited concentration-dependent antioxidant activity [33,34]. Additionally, the IC_50_ values of SR samples were calculated for DPPH, ABTS and *β*-carotene tests, and the results are presented in Supplementary Table S1. The ethyl acetate fraction showed the highest antioxidant activity in ABTS and *β*-carotene tests, and mildly lower activity than the most active n-butanol fraction in the DPPH and FRAP assays. The petroleum ether fraction exhibited the lowest activity in all assays except in the *β*-carotene test. The obtained results were in line with the high total phenolic content and individual compounds found in the n-butanol and ethyl acetate fractions. Many authors have investigated the antioxidant capacity of different *Sideritis* species and reported that the methanol and hydroalcoholic extracts showed higher activity than the ethyl acetate, acetone or dichloromethane extracts [27,28,29,30], which is inconsistent with the results of our study. However, our results are related to the findings of Bardakci et al. [31], who also showed that the ethyl acetate fraction exhibited higher DPPH and FRAP activities than the water, methanol and chloroform fractions, thus pointing out the importance of the successive extraction method for obtaining fractions with different phytochemicals, which then leads to different bioactivity.

Correlation analysis showed that DPPH, ABTS, and FRAP activities are significantly positively correlated with the content of all monitored individual compounds. Generally, the strongest correlations in these assays were reported with ISC2 content followed by HYP2. On the other hand, there were no significant correlations in the case of *β*-carotene antioxidant activity (Table 3). These findings are in agreement with the well-known fact that *β*-carotene activity is contributed mainly by more lipophilic compounds, while the monitored individual compounds were hydrophilic. A similar phenomenon, strong DPPH, ABTS, and FRAP but weak *β*-carotene activity, was also observed in the case of ascorbic acid as a representative hydrophilic antioxidant (Table 2).

### 2.3. Anti-Tyrosinase Activity

The tyrosinase inhibitory activity of hydroethanolic extract and its fractions was tested at three different concentrations (100, 200 and 500 μg/mL), and the results are shown in Table 4. Hydroethanolic extract at all examined concentrations strongly inhibited tyrosinase, and at a concentration of 100 μg/mL, it was even slightly more active (52.64%) than the reference compound, kojic acid (51.81%). Moderate inhibitory activity at a concentration of 100 μg/mL was exhibited by the petroleum ether (34.61%) and ethyl acetate (23.65%) fractions, while water and n-butanol fractions showed weaker anti-tyrosinase activity (20.88% and 16.12%, respectively). However, the inhibitory activity of petroleum ether and water fractions was enhanced with increasing concentration, reaching 51.78% and 45.42% tyrosinase inhibitory activity, respectively. The hydroethanolic extract and most of tested fractions did not inhibit tyrosinase activity in a concentration-dependent manner, as was also noticed in our previous study [35], as well as by other authors [36,37,38]. Contrary to SR samples, kojic acid exhibited concentration-dependent inhibition of tyrosinase, with an IC50 value of 88.94 μg/mL (data not shown). The results obtained indicate that the activity depends on the type of re-extraction solvent used. Although the n-butanol fraction contained the greatest amount of total polyphenols, as well as all individual phenolic compounds, it exhibited relatively low tyrosinase inhibition potency. There were no strong correlations between the content of monitored compounds and anti-tyrosinase activity (Table 3). This may imply that different phytochemical compounds, not detected in our study, such as diterpenoids or hydroxycinnamic acids detected in different *Sideritis* species and known to possesses anti-tyrosinase activity [39,40,41,42], could be responsible for this activity, or that there is a synergism between different classes of secondary metabolites. This can also be connected with the fact that the primary extract, which contains the most diverse group of compounds, which was hydroethanolic extract in our study, showed the best activity.

There have been a few reports of the tyrosinase inhibitory activity of various *Sideritis* extracts [27,28,29,43,44]. Methanol extracts at 200 μg/mL concentration of *S. stricta*, *S. albifora* and *S. leptoclada*, as well as 150 μg/mL methanol extract of *S. sipylea*, showed moderate inhibitory activity, whereas other extracts such as hexane, acetone, water or dichlorometane exhibited low activity or were inactive [27,43,44].

Previous studies on tyrosinase inhibition by compounds isolated from *Sideritis* plants have confirmed the moderate activity (about 2–3-fold lower than kojic acid) of flavonoids and phenylpropanoid glycosides, including verbascoside, as well as slightly lower activity (about 10-fold lower than kojic acid) of phenolic acids, including chlorogenic acid [45]. Tundis et al. [46] reported the strong anti-tyrosinase activity of verbascoside (50% inhibitory concentration of 12.9 µg/mL) isolated from *Stachys lavandulifolia*. In addition to directly inhibiting the enzyme, the verbascoside reduces melanogenesis via down-regulation of tyrosinase protein expression in melanoma cell line B16F10 [39]. The inhibitory activity of verbascoside is related to a free ortho-hydroxyl groups present in the phenolic rings that is capable of chelating the copper ion (Cu^2+^) at the enzyme active site. In a similar manner, flavonoid compounds can build chelate complexes. However, glycone components can reduce the activity by covering the phenolic groups of the aglycone and obstructing the binding with the copper. Thus, enzyme inhibition depends on the position and degree of flavonoid glycosylation. In addition, methoxylation of phenolic groups has also been shown to reduce tyrosinase inhibitory activity [47]. This may partly explain the lower activity of the fractions obtained using organic solvents observed in our study, since methoxylated derivatives have a greater tendency to distribute in nonpolar solvents compared to flavonoids of free phenolic groups that remain in the polar phase. The separation of the mentioned compounds, guided by their lipophilicity, can be observed in the example of the flavonoid ISC2, which showed a higher distribution affinity in organic fractions than compound ISC1.

Our results showed the strongest activity achieved so far against tyrosinase of any plant belonging to the genus *Sideritis*. Moreover, the standard compound kojic acid, as a skin whitening agent, also triggers a variety of side effects, and therefore its usability is limited [45]. In this context, *S. raeseri* extract could be a safer alternative, with a more comprehensive effect on the skin, while maintaining a high anti-tyrosinase potential.

### 2.4. Antimicrobial Activity

The hydroethanolic extract of SR and its fractions were assessed for antibacterial activity against skin infection-causing pathogens *Staphylococcus aureus*, *Staphylococcus epidermidis* and *Pseudomonas aeruginosa*, and the results are shown in Table 5. Antimicrobial activity was detected at comparable levels for all of the investigated samples, with MIC values between 1.88 and 12.42 mg/mL. Both extract and fractions were active against both Gram-positive and Gram-negative bacteria, and the most susceptible was *S. epidermidis*. The ethyl acetate fraction showed the strongest antimicrobial activity, with MIC values of 1.88–3.13 mg/mL, followed by the hydroethanolic extract (3.38–5.08 mg/mL) and the petroleum ether fraction (3.70–6.67 mg/mL). The water fraction exhibited the lowest inhibitory activity (8.68–12.42 mg/mL).

The observed antimicrobial activity of *S. raeseri* extract and its fractions is in accordance with previous research conducted on the aqueous extract of *S. raeseri* and the ethanolic, ethyl acetate and butanol extracts of *S. scardica* [48,49]. These extracts showed moderate activity against *S. aureus*, with MIC values similar to the values obtained in our study. In addition, Sagdic et al. [50] and Ozkan et al. [51] also reported that methanolic extracts of *S. ozturkii*, *S. caesarea* and *S. condensata* exhibited moderate activity against *S. aureus* and *P. aeruginosa*, while *S. eryhrantha* methanolic extract was active against *P. aeruginosa*. According to the literature data, although inhibitory activity against *Staphylococcus epidermidis* has been shown for essential oils of some *Sideritis* species [52,53], there are no reports about such activity of *Sideritis* extracts. This Gram-positive bacterium, previously regarded as a commensal microorganism of the human skin, is now one of the most frequent causes of nosocomial infections similarly as *S. aureus* [54]. Its antibiotic resistance and ability to form biofilms represent a serious treat although it rarely leads to life-threatening diseases. Inhibition of the growth of *S. epidermidis* by our samples used at low concentrations represent a good basis for the development of topical antimicrobial herbal products.

Although the antimicrobial activity of phenolic compounds, particularly flavonoids, has been proven [55], the results of our study were equivocal, since petroleum ether fraction with lowest and ethyl acetate fraction with high total phenolic content displayed good antimicrobial activity, whereas the butanol fraction exerted lower activity despite having the highest total phenolic content. This indicates that this inhibitory activity is due to specific polyphenols or another class of compounds, such as monoterpenoids or diterpenoids, present in the fractions, or due to their synergistic activity. It has been shown that *S. scardica* hexane extract rich in diterpenoids was active against *S. aureus* [56], which may also be the case with our non-polar petroleum ether fraction.

As SR extract and its fractions showed very good antimicrobial activity against *S. epidermidis*, and also against common skin infection bacteria, *S. aureus* and *P. aeruginosa*, which are associated with high antibiotic resistance to the majority of antimicrobials [57], the possibility of using some *S. raeseri* extract or fractions for the topical treatment of skin disorders such as bacterial infections could be considered.

### 2.5. Photoprotective Activity

The SPF values of the SR hydroethanolic extract and its fractions determined at three different concentrations are presented in Table 6. In general, concentration dependence was observed for the SPF value, so that an increase in the sample concentration increased the SPF value. The n-butanol fraction showed the highest SPF values at all tested concentrations (7.81–30.11), followed by ethyl acetate fraction and hydroethanolic extract (5.13–20.31 and 4.54–18.01, respectively), whereas the petroleum ether fraction demonstrated the lowest SPF values (0.99–4.05). According to the recommendations of the European Union Commission (2006/647/EC), sunscreen products should provide SPF ≥ 6 as the minimum degree of UVB protection [58]. All tested samples, with the exception of petroleum ether fraction, met this criterion at a sample concentration of 0.1 mg/mL. This is the first report of photoprotective activity in some *Sideritis* species. El Aanachi et al. [59] measured the SPF of six Moroccan Lamiaceae, and found that methanolic extract of *Salvia officinalis* gave the highest SPF value of 39 at a concentration of 2 mg/mL, which was a concentration 10–20 times higher than those we used in our study.

Taking into account the total phenolic content in hydroethanolic extract of *S. raeseri* and its fractions, the photoprotective activity shown is not surprising, since polyphenols can absorb UV radiation in the range of 200–400 nm, thus acting as good sunscreen pigments [60,61]. Moreover, phenolic compounds, especially flavonoids, have antioxidant properties and can reduce the production of oxygen free radicals caused by UV radiation [62]. In our study, an association was observed between SPF value, total phenolic content and the antioxidant activity of n-butanol and ethyl acetate fractions, suggesting that the photoprotective activity can be attributed to the presence of these compounds.

Finally, correlation analysis shows that the value of the SPF is strongly positively correlated with all observed compounds (Table 6), implying that the compounds jointly contribute to the photoprotective activity.

## 3. Materials and Methods

### 3.1. Chemicals and Reagents

Petroleum ether and acetonitrile were purchased from ThermoFisher Scinetific (Waltham, MA, USA). Ethanol, ethyl acetate, n-butanol, sodium carbonate anhydrous (Na_2_CO_3_) and dimethyl sulfoxide (DMSO) were purchased from Centrohem (Stara Pazova, Serbia). Folin–Ciocalteu reagent and orthophosphoric acid were purchased from Carlo Erba Reagents (Emmendingen, Germany). Galic acid, chlorogenic acid, verbascoside, 2,2-dyphenyl-1-picrylhydrazyl (DPPH), ascorbic acid, 2,2′-azino-bis(3-ethyl-benzothiazoline-6-sulfonic acid) (ABTS), sodium acetate, 2,4,6-tris(2-pyridyl)-(S)-triazine (TPTZ), sodium acetate trihydrate, iron(III) chloride hexahydrate (FeCl_3_ × 6H_2_O), iron(II) sulfate heptahydrate (FeSO_4_ × 7H_2_O), *β*-carotene, linoleic acid, Tween 40, kojic acid, sodium phosphate monobasic, sodium chloride, levodopa (L-DOPA), tyrosinase from mushroom (lyophilized powder, ≥1000 unit/mg solid), streptomycin and resazurin were purchased from Sigma-Aldrich (St. Louis, MO, USA). Butylhydroxyanisole (BHA) and butylhy-droxytoluene (BHT) were purchased from Acros Organics (Geel, Belgium), hydrochloric acid (HCl; 36,2%) from Zorka Pharma (Šabac, Serbia), and chloroform from Macron Fine Chemicals (Center Valley, PA, USA). Isoscutellarein 7-o-[6‴-o-acetyl-*β*-d-allopyranosyl(1→2)]-*β*-d-glucopyranoside (ISC1), 4′-o-methyl-isoscutellarein 7-o-[6‴-o-acetyl-*β*-d-allopyranosyl(1→2)]-*β*-d-glucopyranoside (ISC2), 4′-o-methyl-hypolaetin 7-o-[6‴-o-acetyl-*β*-d-allopyranosyl(1→2)]-*β*-d-glucopyranoside (HYP1), 4′-o-methyl-hypolaetin 7-o-[6‴-o-acetyl-*β*-d-allopyranosyl(1→2)]-6‴-o-acetyl-*β*-d-glucopyranoside (HYP2) were previously isolated [23]. *Staphylococcus aureus* ATCC 29213, *Staphylococcus epidermidis* ATCC 12228 and *Pseudomonas aeruginosa* ATCC 27853 were purchased from Faculty of Biology (Belgrade, Serbia). Mueller Hinton Agar (MHA) and Mueller Hinton Broth (MHB) medium were purchased from Torlak (Belgrade, Serbia).

### 3.2. Plant Material

Aerial parts of *Sideritis raeseri* subsp. *raeseri* (SR) were collected in the full flowering phase in July 2019 at National Park Galičica, North Macedonia. Voucher specimens Nos. S31/16–S33/16 have been deposited at the Institute for Medicinal Plants Research “Dr. Josif Pančić”, Belgrade, Serbia.

### 3.3. Preparation of Extracts

Air-dried and powdered aerial parts (200 g) were extracted with 70% ethanol (1:10, *w/v*) using maceration at room temperature with continuous stirring at 100 rpm, during 24 h. Evaporated residue (27.4 g) was suspended in 100 mL of water and re-extracted successively with petroleum ether (5 × 50 mL), ethyl acetate (5 × 50 mL) and n-butanol (5 × 50 mL), respectively. After filtration, obtained extracts were evaporated under reduced pressure (Buchi rotavapor R-114) till dryness, and 1.6 g of petroleum ether, 1.6 g of ethyl acetate, 3.1 g of n-butanol, and 1.6 g of water fractions were afforded. Extracts were stored at 4 °C for further experiments.

### 3.4. Determination of Total Phenolic Content

The total phenolic content of SR dry hydroethanolic extract and its fractions was estimated by Folin–Ciocalteu method with modifications [63]. Primarily, extracts dissolved in 70% ethanol (200 μL) were mixed with 1:10 water diluted Folin–Ciocalteu reagent (1000 μL). After 4 min, Na_2_CO_3_ solution (75 g/L; 800 μL) was added. The absorbance was measured at 765 nm after incubation for 2 h at room temperature. For the calibration curve preparation, gallic acid standard solutions (0–100 mg/L) were used. The results were expressed as milligrams of gallic acid equivalents per gram of dry weight of extract/fraction (mg GAE/g).

### 3.5. HPLC Analysis

For qualitative and quantitative analyses of SR dry hydroethanolic extract and its fractions, the HPLC (high-performance liquid chromatography) method was applied. The analyses were performed using Agilent 1200 series instrument with DAD detector. Lichrospher RP-18 analytical column (250 × 4 mm i.d., particle size of stationary phases 5 µm) was used for separation. The mobile phase contained solvent A (1% orthophosphoric acid in water) and solvent B (acetonitrile). The following gradient program was used for elution: 90–80% A 0–5 min, 80% A 5–10 min, 80–70% A 10–20 min, 70–30% A 20–30 min, 30–0% A 30–35 min. The injection volume was 5 μL, the flow was 1 mL/min, and detection wavelengths were set at 280 and 330 nm. The sample concentrations were 10–40 mg/mL. Quantification of isoscutellarein 7-o-[6‴-o-acetyl-*β*-d-allopyranosyl(1→2)]-*β*-d-glucopyranoside (ISC1), 4′-o-methyl-isoscutellarein 7-o-[6‴-o-acetyl-*β*-d-allopyranosyl(1→2)]-*β*-d-glucopyranoside (ISC2), 4′-o-methyl-hypolaetin 7-o-[6‴-o-acetyl-*β*-d-allopyranosyl(1→2)]-*β*-d-glucopyranoside (HYP1), 4′-o-methyl-hypolaetin 7-o-[6‴-o-acetyl-*β*-d-allopyranosyl(1→2)]-6‴-o-acetyl-*β*-d-glucopyranoside (HYP2), chlorogenic acid and verbascoside was done using calibration curves of standards. The results were presented as mg/g of dry extract/fraction.

### 3.6. Evaluation of Antioxidant Activity

#### 3.6.1. DPPH Assay

The DPPH (2,2-dyphenyl-1-picrylhydrazyl) assay described by Blois [64] was adopted and used to evaluate radical scavenging activity of the SR hydroethanolic extract and its fractions. 100 μL of samples dissolved in ethanol with different concentrations (100, 200 and 500 μg/mL) and DPPH ethanol solution (40 µg/mL) (900 μL) were mixed and incubated in the dark for 30 min, at room temperature. Ascorbic acid, butylhydroxyanisole (BHA), and butylhydroxytoluene (BHT) were used as positive controls. The absorbance was measured at 517 nm. Inhibition of DPPH radicals was calculated using the following formula:

Inhibition of DPPH radical (%) = [(A_C_ − A_S_)/A_C_] × 100 (1)

where A_C_ is the absorbance of the control and A_S_ is the absorbance of the tested samples.

#### 3.6.2. ABTS Assay

Modified procedure of Miller et al. [65] for ABTS (2,2′-azino-bis(3-ethyl-benzothiazoline-6-sulfonic acid) assay was used to examine radical scavenging activity of the SR hydroethanolic extract and its fractions. First, 25 μL of samples dissolved in ethanol with different concentrations (100, 200 and 500 μg/mL), as well as positive controls (ascorbic acid, BHA and BHT), were mixed with properly prepared ABTS^•+^ solution (1000 μL), and incubated at 30 °C for 30 min. Absorbance was recorded at 734 nm. The inhibition of ABTS radical was calculated using the following formula:

Inhibition of ABTS radical (%) = [(A_C_ − A_S_)/A_C_] × 100 (2)

where A_C_ is the absorbance of the control and A_S_ is the absorbance of the samples.

#### 3.6.3. FRAP Assay

The total antioxidant capacity of the SR hydroethanolic extract and its fractions was investigated by ferric reducing antioxidant power (FRAP) assay. The procedure for the FRAP assay of Benzie and Strain [66], with some modifications previously described by Šavikin et al. [35], was applied. FRAP reagent, freshly prepared, contained 10 mL of sodium acetate buffer (300 mmol/L, pH 3.6), 1 mL of 10 mmol/L 2,4,6-tris(2-pyridyl)-(S)-triazine (TPTZ) in 40 mmol/L HCl and 1 mL of FeCl_3_ × 6H_2_O solution (20 mmol/L). Samples were dissolved in ethanol. Ascorbic acid, BHA, and BHT were used as positive controls. Working FRAP reagent (1200 μL) and sample/positive control solution (40 μL) were mixed, and heated to 37 °C. After 4 min, absorbance was recorded at 593 nm. Results were expressed as μmol FeSO_4_ × 7H_2_O/g of dry extract or fraction.

#### 3.6.4. *β*-Carotene Bleaching Assay

The antioxidant activity of the SR hydroethanolic extract and its fractions against lipid peroxyl radical in *β*-carotene bleaching assay was measured according to the procedure of Dapkevicius et al. [67]. Samples dissolved in ethanol (140 μL) were mixed with *β*-carotene/linoleic acid emulsion (1000 μL), prepared by dissolving *β*-carotene (1 mg), linoleic acid (50 μL) and Tween 40 (400 mg) in 2 mL of chloroform. The absorbances were measured immediately and after 120 min incubation at 490 nm. Ascorbic acid, BHA, and BHT were used as positive controls. The percentage of *β*-carotene oxidative degradation inhibition was calculated using the following formula:

Inhibition (%) = [(A_120_ − C_120_)/(C_0_ − C_120_)] × 100 (3)

where A_120_ (samples) and C_120_ (control) are the absorbances measured after 120 min incubation, while C_0_ (control) is the absorbance measured immediately.

### 3.7. Tyrosinase Inhibitory Activity Assay

The spectrophotometric method described by Masuda et al. [68] was used for evaluation of SR hydroethanolic extract and its fractions tyrosinase inhibitory activity. Kojic acid (positive control) and samples were dissolved in sodium phosphate buffer (0.1 M, pH 7.0), which contained 5% dimethyl sulfoxide (DMSO), while solutions of tyrosinase (40 u/mL) and substrate levodopa (L-DOPA), (2.5 mM) were prepared in sodium phosphate buffer (0.1 M, pH 7.0). The experiment was done in a 96-well microplate. After the addition of 40 μL of L-DOPA to C (control: sodium buffer + tyrosinase), S (sample: buffer + tyrosinase + sample), and B (blank: buffer + sample), and incubation at 25 °C for 30 min, the absorbance was measured at 475 nm. The percentage inhibition of tyrosinase activity was calculated using the following formula:

Inhibition (%) = [A_C_ − (A_S_ − A_B_)/A_C_] × 100 (4)

where A_B_ and A_C_ are absorbances of blank and control solutions, respectively, while A_S_ is the absorbance of the samples.

### 3.8. Antimicrobial Assay

The antibacterial activity of SR hydroethanolic extract and its fractions was screened using the broth microdilution method to determine the minimum inhibitory concentration (MIC) against three bacteria, the most common cause of skin infections in humans: two Gram positive bacteria *Staphylococcus aureus* ATCC 29213 and *Staphylococcus epidermidis* ATCC 12228, and *Pseudomonas aeruginosa* ATCC 27853 as Gram negative. The test was performed according to the recommendations of the Clinical Laboratory Standard Institute [69]. Bacterial species were sub-cultured on Mueller Hinton Agar (MHA) (pH 7.2), incubated at 37 °C for 24 h, and then cultured overnight at 37 °C in Mueller Hinton Broth (MHB) medium. The bacterial culture was adjusted to an optical density 1 × 10^8^ CFU/mL using a DEN-1B McFarland Densitometer (Biosan, Latvia); afterwards, bacterial cell suspensions were adjusted with sterile saline to a final concentration of inoculum (5 × 10^5^ CFU/mL) in each well. The inocula were stored at 4 °C. To verify the absence of contamination and to check the validity of the inoculum, dilutions of the inocula were cultured on solid MHA.

Determination of MICs was performed by a serial dilution technique using 96-well microtiter plates. Tested samples were dissolved in dimethyl sulfoxide (DMSO) and added to MHB medium with inoculum. Serial dilutions of the working solution were prepared by diluting the extract solution in sterile MHB dispensed to all wells. A working solution of extract and its fractions and solvent controls (MHB and DMSO) were dispensed into their respective wells. Serial dilutions were performed from columns 1 to 9, and 50 μL of excess medium was discarded from column 9. The last column served as a blank, which contained only broth. Columns 10 and 11 served as negative controls, which only contained medium and bacterial suspension, and media DMSO and bacteria, respectively. Test bacteria were added to each well, except for the last row, which served as a blank. Streptomycin was used as positive control.

The plates were sealed and incubated for 24 h at 37 °C. After 24 h incubation, resazurin was added to all wells, and incubated at 37 °C for 30 min. The MIC of the samples was detected after 30 min of incubation. The wells containing the bacterial growth turned into pink color, whereas the well without bacterial growth remained blue. MIC was defined as the sample concentration that prevented the color change of the medium and exhibited complete inhibition of microbial growth.

### 3.9. Photoprotective Assay

The photoprotective capacity of SR hydroethanolic extract and its fractions was determined spectrophotometrically according to the method described by Oliveira et al. [70]. As a universal measure of photoprotective activity, the sun protection factor (SPF) was calculated. Solutions of examined samples at concentrations of 0.05, 0.1 and 0.2 mg/mL were prepared in phosphate buffer (pH 7.0). Absorption of each solution was measured in triplicate at 290–320 nm, with 5 nm increments, using Agilent Cary 3500 UV-Vis Spectrophotometer. Phosphate buffer was used as a blank. SPF was calculated using the following formula:

(5) $$\mathrm{SPF}=\mathrm{CF}\times \overset{320}{\underset{290}{\sum }}\mathrm{EE}\left(\lambda \right)I\left(\lambda \right)\mathrm{Abs}\left(\lambda \right)$$

where CF is the correction factor (equal to 10); EE (λ) is the erythemal effect spectrum; I (λ) is the solar intensity spectrum, and Abs (λ) is the absorbance of the solution at a wavelength (λ). The values of EE × I are constants, and were determined by Sayre et al. [71].

### 3.10. Statistical Analysis

All analyses were performed in triplicate, and the results are expressed as mean ± standard deviation. One-way analysis of variance (ANOVA) with Tukey’s post hoc test was used to identify statistically significant differences (*p* < 0.05) and for multiple comparisons. Relationships between the content of individual compounds in extract/fractions and their bioactivities were measured using the Pearson correlation analysis with a statistical significance level of 0.05. Statistical analysis was performed using a trial version of the SPSS software package (Dublin, Ireland).

## 4. Conclusions

The aim of our study was to perform an extraction and fractionation method that would result in the recovery of active compounds from *Sideritis raeseri*, and to test biological activity of the obtained fractions. We used 70% ethanol for the sequential extraction of phenolic compounds, as they are easily solubilized in hydroalcoholic mixture and the fractionation was performed using petroleum ether, ethyl acetate, n-butanol, and water in order to enable the separation of the constituents with respect to their polarity. The obtained fractions were tested for different biological activities, namely antioxidant, anti-tyrosinase, antimicrobial and photoprotective activity. The results showed that ethyl acetate and n-butanol fractions, the richest in total phenolic content, were the most active in antioxidant (*β*-carotene, DPPH, ABTS, FRAP) assays. The best antimicrobial activity against *Staphylococcus aureus*, *Staphylococcus epidermidis* and *Pseudomonas aeruginosa*, the most common causative agents of skin infections in humans, was achieved by the ethyl acetate fraction. Photoprotective activity (SPF > 6) was shown for almost all fractions, but the most promising was the butanol fraction with SPF > 10 and SPF > 30, obtained using 0.1 and 0.2 mg/mL of the butanol fraction, respectively. The hydroethanolic extract and petroleum ether fractions were strong anti-tyrosinase agents. Our findings indicate the importance of the successive extraction method for obtaining fractions that exert diverse biological effects due to the presence of different classes of compounds. The application of this method of extraction makes it possible to obtain *S. raeseri* fractions with a broad spectrum of bioactivities, enabling this plant to be a beneficial multi-target ingredient for various formulations.

## Figures and Tables

**Table 1:** Content of total phenolic and individual compounds in *Sideritis raeseri* subsp. *raeseri* aerial parts hydroethanolic extract and its fractions.

Sample	Total Phenolic Content (mg GAE/g)	Individual Compounds (mg/g)					
		ISC1	ISC2	HYP1	HYP2	Chlorogenic Acid	Verbascoside
**Hydroethanolic extract**	48.25 ± 1.44 ^b^	9.57 ± 0.33 ^d^	16.82 ± 0.39 ^c^	15.46 ± 0.32 ^c^	3.97 ± 0.09 ^c^	1.88 ± 0.02 ^c^	8.20 ± 0.04 ^c^
**Petroleum ether fraction**	13.45 ± 1.08 ^a^	0.67 ± 0.03 ^a^	2.83 ± 0.05 ^a^	1.95 ± 0.05 ^a^	0.31 ± 0.01 ^a^	0.21 ± 0.01 ^a^	1.62 ± 0.01 ^a^
**Ethyl acetate fraction**	88.48 ± 1.29 ^c^	5.41 ± 0.20 ^b^	26.15 ± 0.66 ^d^	5.77 ± 0.16 ^b^	10.62 ± 0.27 ^d^	0.93 ± 0.02 ^b^	16.35 ± 0.03 ^d^
**Butanol fraction**	92.20 ± 0.50 ^d^	27.14 ± 0.72 ^e^	32.4 ± 0.79 ^e^	26.47 ± 0.54 ^d^	11.9 ± 0.22 ^e^	2.85 ± 0.03 ^d^	31.01 ± 0.01 ^e^
**Water fraction**	46.65 ± 1.18 ^b^	8.03 ± 0.30 ^c^	12.98 ± 0.23 ^b^	15.49 ± 0.34 ^c^	2.49 ± 0.04 ^b^	1.84 ± 0.02 ^c^	4.88 ± 0.01 ^b^

Different letters (a–e) in the same columns indicate a significant difference (*p* < 0.05) in content between extract and its fractions according to the *post hoc* Tukey’s test. ISC1: Isoscutellarein 7-o-[6‴-o-acetyl-*β*-d-allopyranosyl-(1→2)]-*β*-d-glucopyranoside;.ISC2: 4′-o-methyl-Isoscutellarein 7-o-[6‴-o-acetyl-*β*-d-allopyranosyl-(1→2)]-*β*-d-glucopyranoside;.HYP1: 4′-o-methyl-Hypolaetin 7-o-[6‴-o-acetyl-*β*-d-allopyranosyl (1→2)]-*β*-d-glucopyranoside;.HYP2: 4′-o-methyl-Hypolaetin 7-o-[6‴-o-acetyl-*β*-d-allopyranosyl (1→2)]-6‴-o-acetyl-*β*-d-glucopyranoside.

**Table 2:** Antioxidant activity of the hydroethanolic extract of the aerial parts of *Sideritis raeseri* subsp. *raeseri* and its fractions.

Sample	Concentration [µg/mL]	*β*-Carotene [% Inhibition]		DPPH [% Inhibition]		ABTS [% Inhibition]		FRAP [µmol Fe (II)/g]	
Hydroethanolic extract	100 200 500	30.12 ± 0.39 ^a^ 33.77 ± 1.26 ^b^ 38.33 ± 0.78 ^c^	d	8.26 ± 0.79 ^a^ 17.51 ± 0.97 ^b^ 45.22 ± 0.53 ^c^	b	8.14 ± 0.59 ^a^ 18.32 ± 0.69 ^b^ 34.37 ± 0.37 ^c^	c	140.72 ± 5.18 ^a^ 167.70 ± 5.71 ^b^ 389.79 ± 3.80 ^c^	c
Petroleum ether fraction	100 200 500	19.69 ± 1.48 ^a^ 22.69 ± 1.03 ^a^ 36.51 ± 1.48 ^b^	c	2.67 ± 0.32 ^a^ 4.99 ± 0.70 ^b^ 6.96 ± 0.32 ^c^	a	1.75 ± 0.22 ^a^ 3.05 ± 0.44 ^b^ 6.54 ± 0.59 ^c^	a	32.38 ± 3.13 ^a^ 52.30 ± 3.13 ^b^ 74.72 ± 4.00 ^c^	a
Ethyl acetate fraction	100 200 500	28.55 ± 1.17 ^a^ 34.94 ± 0.60 ^b^ 41.07 ± 0.78 ^c^	d	12.31 ± 0.48 ^a^ 26.86 ± 0.91 ^b^ 69.41 ± 0.63 ^c^	d	22.39 ± 0.51 ^a^ 37.37 ± 0.47 ^b^ 57.44 ± 0.80 ^c^	e	132.84 ± 3.74 ^a^ 353.67 ± 5.03 ^b^ 501.87 ± 3.80 ^c^	c
Butanol fraction	100 200 500	2.50 ± 2.10 ^a^ 7.09 ± 1.20 ^b^ 13.21 ± 1.50 ^c^	a	13.71 ± 0.38 ^a^ 45.99 ± 0.53 ^b^ 91.60 ± 0.78 ^c^	e	13.96 ± 0.47 ^a^ 29.96 ± 0.37 ^b^ 50.36 ± 0.37 ^c^	d	137.82 ± 3.29 ^a^ 354.50 ± 6.93 ^b^ 603.99 ± 5.18 ^c^	c
Water fraction	100 200 500	7.93 ± 0.64 ^a^ 8.90 ± 1.93 ^a^ 17.39 ± 0.83 ^b^	b	10.48 ± 0.75 ^a^ 15.40 ± 0.97 ^b^ 45.04 ± 0.63 ^c^	c	5.04 ± 0.67 ^a^ 13.33 ± 0.25 ^b^ 25.40 ± 0.44 ^c^	b	61.44 ± 4.37 ^a^ 129.10 ± 4.98 ^b^ 278.95 ± 5.18 ^c^	b
BHA	100	57.70 ± 1.91	e	43.33 ± 0.87	g	64.95 ± 0.63	g	572.85 ± 5.71	e
BHT	100	56.29 ± 1.44	e	34.31 ± 0.43	f	55.11 ± 0.44	f	413.03 ± 3.13	d
Ascorbic acid	100	2.99 ± 2.13	a	91.65 ± 0.21	h	55.70 ± 0.69	f	576.17 ± 7.61	e

Different letters (a–c) within the same cells indicate a significant difference (*p* < 0.05) in activity between different tested concentrations while different letters (a–h) in the same columns indicate a significant difference in activity between extract/fractions and standards at the concentration of 100 μg/mL according to the *post hoc* Tukey’s test.

**Table 3:** Correlation between the content of the target compounds of the tested extract and fractions and biological activity, expressed by the Pearson’s coefficient.

	*β*-Carotene	DPPH	ABTS	FRAP	Anti-Tyrosinase	SPF
**ISC1**	−0.42	0.78 **	0.55*	0.69 *	−0.44	0.80 **
**ISC2**	0.12	0.99 **	0.93 **	0.97 **	−0.49	0.98 **
**HYP1**	−0.19	0.84 **	0.61 *	0.76 **	−0.13	0.89 **
**HYP2**	0.11	0.94 **	0.93 **	0.94 **	−0.64 *	0.90 **
**Chlorogenic acid**	−0.12	0.87 **	0.67 *	0.80 **	−0.14	0.79 **
**Verbascoside**	−0.19	0.93 **	0.83 **	0.90 **	−0.57 *	0.90 **

* *p* < 0.05; ** *p* < 0.01; SPF—sun protection factor.

**Table 4:** Anti-tyrosinase activity of the hydroethanolic extract of the aerial parts of *Sideritis raeseri* subsp. *raeseri* and its fractions.

Sample	Concentration [µg/mL]	Tyrosinase [% Inhibition]
Hydroethanolic extract	100 200 500	52.64 ± 0.46 ^a^ 50.05 ± 1.58 ^a^ 49.75 ± 2.95 ^a^
Petroleum ether fraction	100 200 500	34.61 ± 1.27 ^a^ 47.03 ± 3.16 ^b^ 51.78 ± 4.78 ^b^
Ethyl acetate fraction	100 200 500	23.65 ± 2.76 ^b^ 4.29 ± 1.27 ^a^ 6.48 ± 1.67 ^a^
Butanol fraction	100 200 500	16.12 ± 3.36 ^b^ 20.51 ± 2.29 ^b^ 9.52 ± 2.29 ^a^
Water fraction	100 200 500	20.88 ± 4.40 ^a^ 40.66 ± 2.91 ^b^ 45.42 ± 4.44 ^b^
Kojic acid	100	51.81 ± 2.55

Different letters (a,b) within the same cells indicate a significant difference (*p* < 0.05) between different tested concentrations according to the post hoc Tukey’s test.

**Table 5:** Antibacterial activity of the hydroethanolic extract of the aerial parts of *Sideritis raeseri* subsp. *raeseri* and its fractions.

Sample	Minimal Inhibitory Concentrations [mg/mL]		
	*Staphylococcus aureus*	*Staphylococcus epidermidis*	*Pseudomonas aeruginosa*
Hydroethanolic extract	5.08 ± 0.61 ^c^	3.38 ± 0.23 ^c^	4.85 ± 0.20 ^c^
Petroleum ether fraction	4.67 ± 0.15 ^c b^	3.70 ± 0.41 ^c^	6.67 ± 0.53 ^d^
Ethyl acetate fraction	3.13 ± 0.28 ^b^	1.88 ± 0.30 ^b^	2.51 ± 0.32 ^b^
Butanol fraction	7.50 ± 0.31 ^d^	5.03 ± 0.23 ^d^	8.75 ± 0.50 ^e^
Water fraction	12.42 ± 1.16 ^e^	8.68 ± 0.55 ^e^	11.18 ± 0.72 ^f^
Streptomycin	0.09 ± 0.01 ^a^	0.03 ± 0.00 ^a^	0.08 ± 0.01 ^a^

Different letters (a–f) indicate a significant difference (*p* < 0.05) in activity between extract and its fractions according to post hoc Tukey’s test.

**Table 6:** Sun protection factor (SPF) of the hydroethanolic extract of the aerial parts of *Sideritis raeseri* subsp. *raeseri* and its fractions.

Concentration [mg/mL]	SPF				
	Hydroethanolic Extract	Petroleum Ether Fraction	Ethyl Acetate Fraction	Butanol Fraction	Water Fraction
0.05	4.54 ± 0.05 ^c^	0.99 ± 0.01 ^a^	5.13 ± 0.02 ^d^	7.81 ± 0.02 ^e^	4.07 ± 0.03 ^b^
0.1	9.33 ± 0.13 ^c^	2.16 ± 0.01 ^a^	10.18 ± 0.10 ^d^	15.27 ± 0.10 ^e^	8.08 ± 0.04 ^b^
0.2	18.01 ± 0.08 ^c^	4.05 ± 0.03 ^a^	20.31 ± 0.10 ^d^	30.11 ± 0.12 ^e^	16.11 ± 0.12 ^b^

Different letters (a–e) indicate a significant difference (*p* < 0.05) between extract and its fractions according to the *post hoc* Tukey’s test.

## Data Availability

Not applicable.

## Supplementary Materials

The following supporting information can be downloaded at: https://www.mdpi.com/article/10.3390/plants11202677/s1, Table S1: IC50 values (μg/mL) of SR samples in DPPH, ABTS and β-carotene bleaching assays.
