# Supplementary material for: Bioassay-Guided Skin-Beneficial Effects of Fractionated Sideritis raeseri subsp. raeseri Extract
Source: Plants (Basel). 2022 Oct 11;11(20):2677. doi: 10.3390/plants11202677 (PMC9609566; doi:10.3390/plants11202677)
Supplement: Supplementary file 1 [file plants-11-02677-s001.zip › plants-1893493-supplementary.pdf]

plants

Supplementary Material

Table S1. IC50 values (μg/mL) of SR samples in DPPH, ABTS and *β*-carotene bleaching assays.

Sample	IC50 value (μg/mL)		
	***β*-carotene bleaching assay**	**DPPH assay**	**ABTS assay**
Hydroethanolic extract	>1000	551.63	739.83
Petroleum ether fraction	817.84	>1000	>1000
Ethyl acetate fraction	792.40	363.63	398.79
Butanol fraction	>1000	264.26	483.40
Water fraction	>1000	561.76	998.46
BHA	50.56	123.40	52.05
BHT	64.43	165.41	85.78
Ascorbic acid	>1000	41.83	86.27
